# In-human clinical experience with direct stick embolization of low-flow vascular malformations using a mammalian target of rapamycin inhibitor

**DOI:** 10.1016/j.jvsv.2024.101997

**Published:** 2024-11-06

**Authors:** Valentina Restrepo-Espinosa, Alfred I. Lee, Stephanie Prozora, Prashant Patel, Naiem Nassiri

**Affiliations:** a Hematology Section, Yale School of Medicine, New Haven, CT; b Hematology Program, Yale New Haven Health, New Haven, CT; c Investigational Drug Service, Yale New Haven Health, New Haven, CT; d The Vascular Care Group (TVCG), Darien, CT; e Vascular Anomalies and Malformations Program (VAMP), Yale School of Medicine, New Haven, CT; f Division of Vascular Surgery, Department of Surgery, Yale New Haven Health, New Haven, CT

**Keywords:** Vascular malformations, Venous malformations, Lymphatic malformations, Klippel-Trenaunay syndrome, Direct stick embolization, mTOR, Sirolimus, Rapamycin

## Abstract

**Background:**

Although direct stick embolization (DSE) of low-flow vascular malformations (LFVMs) with off-label embolotherapeutic compounds is the current mainstay of therapy, systemic oral mammalian target of rapamycin (mTOR) inhibition has evolved into an important adjunctive therapy that is associated with frequent blood draws, systemic toxicity, and rebound signs and symptoms upon cessation. We herein report our experience with in-human DSE of LFVMs with an mTOR inhibitor for direct, intralesional targeting of the culprit mutated pathway without repeated systemic exposure.

**Methods:**

Since 2020, 33 procedures involving DSE were performed in 25 patients with LFVMs using a patented formulation and technique involving the intravenously compatible mTOR inhibitor Yale-OCR7737, used as a liquid compound in a collagen matrix emulsion for added viscosity and intralesional residence. Data were maintained prospectively and reviewed retrospectively for technical success (successful catheterization of the lesion and intralesional delivery of compound), clinical success (improvement in signs/symptoms with radiologically documented reduction in flow and/or volume of treated lesion), complications, side effects, and reinterventions.

**Results:**

From 2020 to 2023, 33 procedures involving DSE were performed using Yale-OCR7737 in 25 patients (10 men [40%]; 15 women [60%]; mean age, 28 years [range, 1-70 years]) with LFMVs involving the head/neck (48%) and limbs (40%); 88% were nonsyndromic and 12% had Klippel-Trenaunay syndrome; 68% exhibited venous malformations, and 32% had lymphatic malformations. Technical and clinical success rates were 100%. Mean DSE sessions per patient was 1.4 (range, 1-5). Localized intravascular coagulopathy was present after 16 DSE procedures (49%); D-dimer improved after DSE in 7 cases. No perioperative or delayed complications occurred. Side effects were seven cases (21%) of self-limited, transient, oral aphthous ulcers.

**Conclusions:**

Our findings suggest that DSE of LFVMs with mTOR inhibitors (Yale-OCR7737) may be safe and effective. This may represent the new embolotherapeutic frontier in the endovascular treatment of LFMVs.

**Article Highlights**

• **Type of Research:** Single-center retrospective analysis of prospectively collected clinical data
• **Key Findings:** Direct stick embolization of low-flow vascular malformations using a mammalian target of rapamycin (mTOR) inhibitor in 25 patients resulted in a clinical and technical success rate of 100%, no perioperative or delayed complications, and a side effect profile limited to seven cases of transient, self-limited aphthous ulcers.
• **Take Home Message:** Direct stick embolization with this mTOR inhibitor may be safe and effective for the management of low-flow vascular malformations.

Vascular malformations are structurally abnormal blood vessels and are classified broadly by the International Society for Study of Vascular Anomalies as high-flow or low-flow lesions.^1^^,^^2^ Low-flow vascular malformations (LFVMs) affect 1 out of every 1000 live births and VMs are the most common type of vascular malformation, with a prevalence of 1% in the general population.^3^^,^^4^ LFVMs are usually caused by sporadic, gain-of-function mutations that affect the PI3K/AKT/mammalian target of rapamycin (mTOR) pathway, which is involved in angiogenesis, cell growth, proliferation, and apoptosis.^1^

The clinical manifestations of LFVMs vary widely and depend on the extent and location of the vascular malformations. Small superficial lesions may only present with minor pain and disfigurement, whereas extensive lesions involving the muscles, bones, and viscera can lead to significant pain, functional impairment, disfigurement, infection and, in the case of VMs, localized intravascular coagulopathy (LIC) or disseminated intravascular coagulation, which may present as life-threatening bleeding and/or thromboembolism.^5^ Because they do not regress or involute spontaneously or independently throughout the course of the patient's lifespan, LFVMs are permanent pathological fixtures that can in fact enlarge and become more symptomatic over time owing to a variety of environmental factors, such as fluctuations in the hormonal milieu, repetitive motion and activity, and trauma.^6^ Large VMs may even lead to bony hypoplasia and demineralization, which is especially problematic in growing children.^7^ Furthermore, skeletal overgrowth and limb length and girth asymmetry are pathognomonic features of many syndromic varieties of LFVMs, such as Klippel Trenaunay syndrome and congenital lipomatous overgrowth with vascular anomalies, epidermal nevus and skeletal deformities syndrome.^8^, ^9^, ^10^

For decades, direct stick embolization (DSE), also referred to as sclerotherapy, and surgical excision have been the two mainstays of treatment for LFVMs. DSE is used more widely, with various techniques and embolic agents used. All embolic agents are used off label, and no target the PI3K/AKT/mTOR pathway mutations are responsible for LFVMs. The most potent sclerosant is ethanol, but its use is limited owing to the potential for severe systemic and localized complications, including cardiovascular collapse and death.^6^^,^^8^^,^^11^ Moreover, extensive VMs may recur regardless of the embolic agent used.^6^ Foamed detergents, such as sodium tetradecyl sulfate and polidocanol, are less effective but also less toxic, making them more widely used. Because the techniques and embolic agents used vary widely without standardization of embolotherapeutic protocols, determining the reported outcomes, treatment efficacy, and complication rates is challenging based on current literature.^8^

Overall, current treatment options for LFVMs, including systemic pharmacotherapy and DSE, are rarely curative.^8^^,^^9^^,^^12^ Fortunately, the discovery of the mutated metabolic pathway responsible for LFVMs has opened the door for a new era of targeted therapies.^1^ The main targeted drug used for the management of LFVMs is sirolimus, also called rapamycin, which is an mTOR inhibitor.^1^ Phase 1 and 3 clinical trials have shown its efficacy in decreasing lesion size and improving symptoms related to LFVMs, while also reporting its wide array of systemic side effects, which include bone marrow toxicity, mucositis, hypercholesterolemia, and elevated liver enzymes.^12^, ^13^, ^14^, ^15^, ^16^, ^17^, ^18^

The efficacy of oral systemic mTOR inhibition for the management of LFVMs reported in these landmark studies led us to seek an alternative route of administration that allowed harnessing its benefits while minimizing its systemic absorption and side effects. Direct intralesional delivery offers this possibility. Currently, there are no mTOR inhibitor formulations available for intralesional delivery through DSE. Therefore, we repurposed, reformulated, and patented a sterile, intravenous compatible mTOR inhibitor formulation, called Yale-OCR7737, for use as an embolotherapeutic agent for DSE of LFVMs. Herein, we report this first-in-human clinical experience with 33 DSE procedures using Yale-OCR7737 in 25 patients with LFVMs.

## Methods

From 2020 to 2023, 33 procedures involving DSE were performed using Yale-OCR7737 in 25 patients with LFVMs. Yale-OCR7737 represents a sterile, US Food and Drug Administration-approved, intravenous mTOR inhibitor formulation prepared by the in-patient surgical compound pharmacy for a patented novel application, concentration, and route of delivery. The sterile liquid medication is then prepared as an emulsion by mixing and agitating it with a total of 5 to 10 mL gelatin matrix along with 2 mL of iodinated contrast for radio-opacity during fluoroscopic delivery. The final concentration ranged between 1 and 2 mg/mL, and the volume injected was entirely dependent on angiographic findings. This emulsion is then delivered per usual, standard DSE protocol as has been previously described.^8^ All data were maintained prospectively and reviewed retrospectively for technical success (successful endoluminal catheterization and intralesional delivery of compound), clinical success (improvement in signs/symptoms [requiring a two-clinician verification process, one surgical and one medical] with radiologically documented reduction in flow and/or volume of treated lesion), complications, side effects, and reinterventions. Institutional review board approval was obtained for access to patient records and the collection of clinical outcomes data.

### Preoperative imaging

Our standard protocol was to perform a preprocedural contrast-enhanced magnetic resonance imaging (MRI) of the lesion. This included triplanar T2-weighted imaging with fat saturation, axial T1-weighted imaging before the administration of radiocontrast dye, and T1-weighted imaging with fat saturation after the administration of radiocontrast dye. In addition, all patients underwent preoperative Duplex ultrasound examination with B-mode imaging, color flow Doppler imaging, and spectral waveform analysis, all of which are modalities that—along with clinical examination and MRI images—were used to confirm a LFVM diagnosis.

### Preoperative laboratory investigation

**Fig 1:**
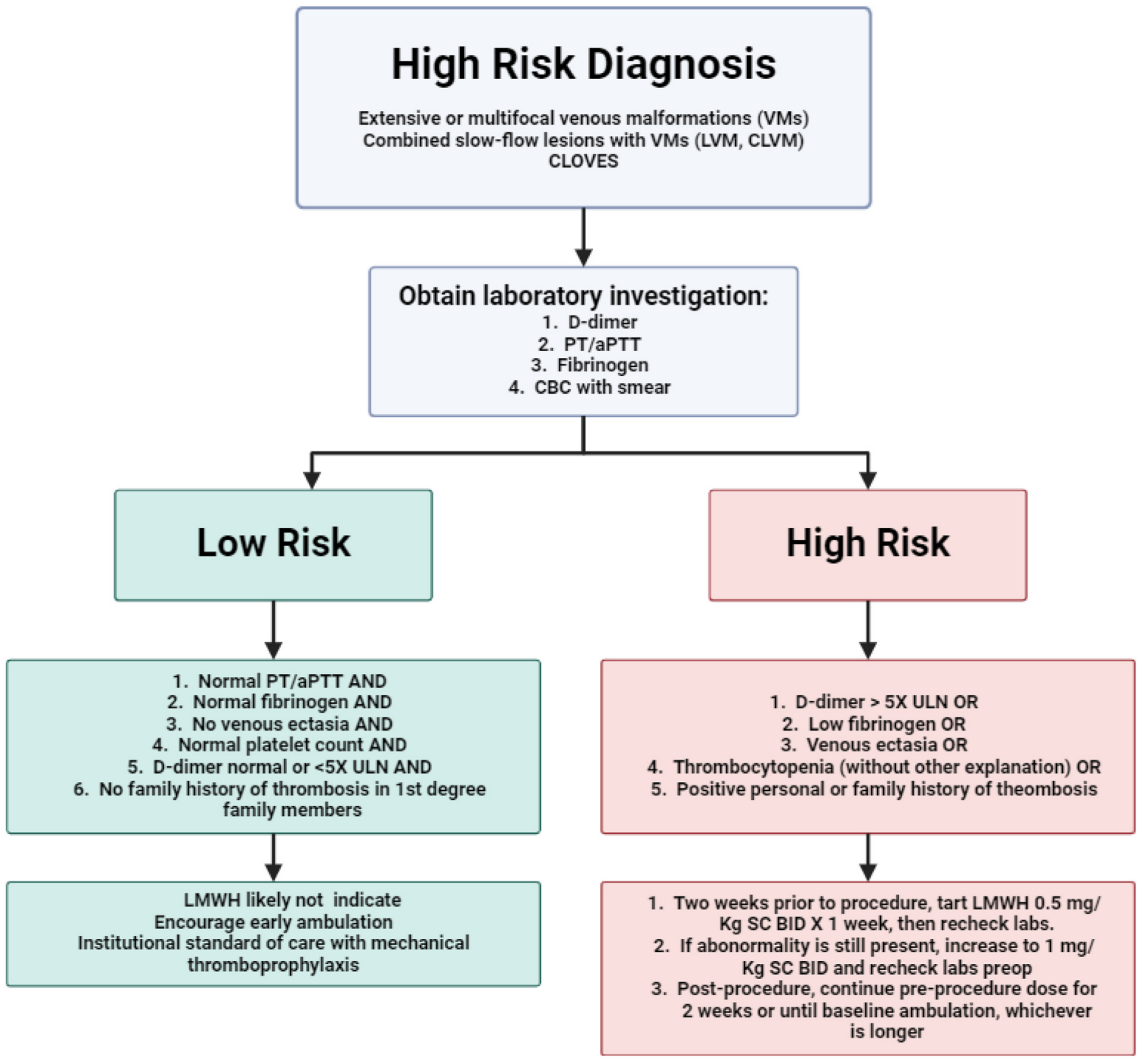
Algorithm delineating perioperative anticoagulation for patients with low-flow vascular malformations (LFVMs) undergoing direct stick embolization (DSE). This is an adaptation of our previously published algorithm (Nassiri et al^19^). Patients who will undergo DSE must be assessed according to their clotting risk to determine the need or perioperative anticoagulation. *aPTT*, activated partial thromboplastin time; *BID*, 2 times a day; *CLOVES*, congenital lipomatous overgrowth with vascular anomalies, epidermal nevus, and skeletal deformities; *CLVM*, capillary lymphatic venous malformation (KTS); *LVM*, lymphatic venous malformation; *PT*, prothrombin time; *SC*, subcutaneous; *ULN*, upper limit of normal.

In all patients with VMs, we monitored coagulation parameters, fibrinogen, and D-dimer levels. According to these results and patient characteristics, we decided if we needed to provide prophylaxis and maintenance anticoagulation for each patient. Our protocol for this decision-making process has been previously reported (Fig 1) and was followed in all these cases accordingly.

### DSE procedure

**Fig 2:**
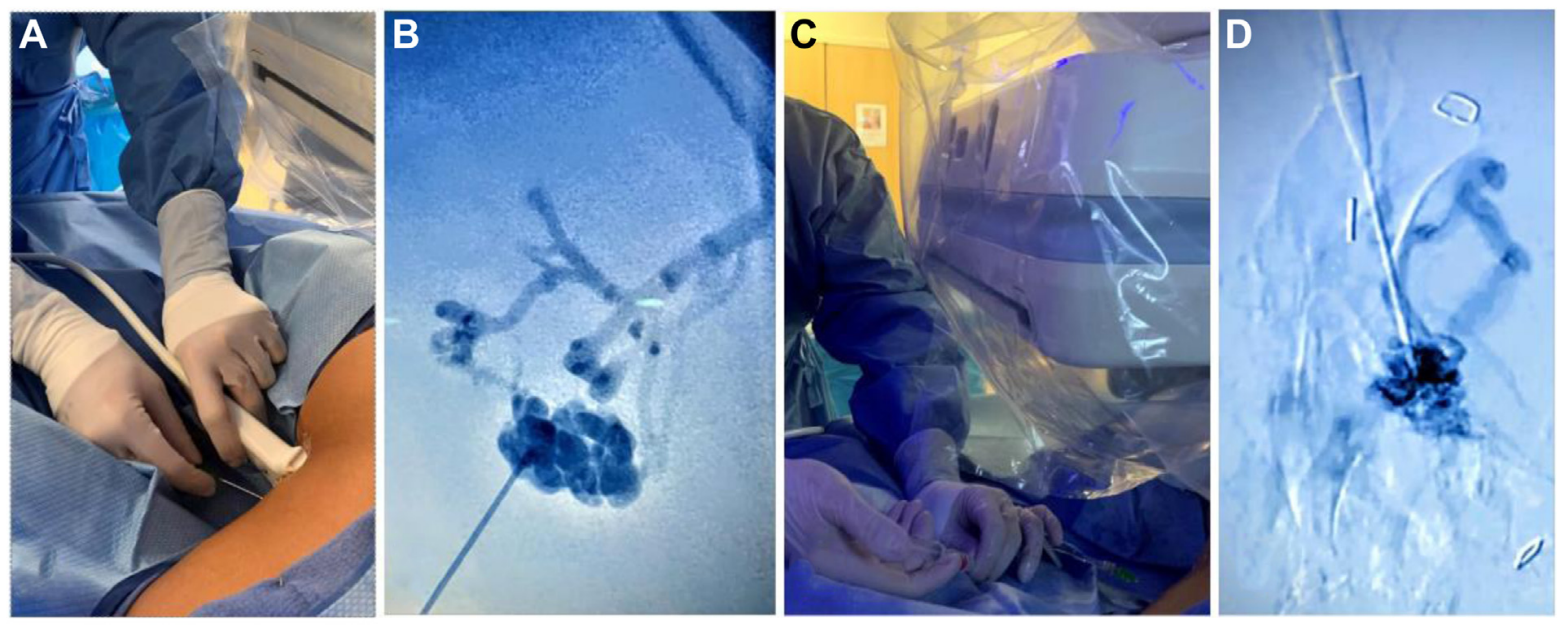
Steps taken during each direct stick embolization (DSE) procedure. Sequence of images depicting the process to perform each DSE. **(A)** Using ultrasound guidance, a needle sheath combination is used to access the low-flow vascular malformation (LFVM). **(B)** Selective venography is done to confirm the intraluminal placement of the catheter and assess the anatomical configuration of the LFVM. **(C** and **D)** We infused a mixture of Yale-OCR7737, Surgiflo, and 1 part air, directly into the vascular channel under direct fluoroscopic visualization.

All DSE procedures were performed by a single operator (NN) using the described formulation and technique involving the intravenously compatible mTOR inhibitor Yale-OCR7737. Ultrasound-guided or blind percutaneous access into the foci of LFVMs was performed using angiocatheters or micropuncture needles, depending on the depth of the lesion (Fig 2, *A*). Selective venography was then performed to delineate the targeted lesion's angioarchitecture and to confirm intraluminal access without extravasation (Fig 2, *B*). For lymphatic malformations, percutaneous drainage of lymphatic contents preceded pre-embolization lymphangiography. Once the angioarchitecture and catheter location were confirmed, we proceeded with direct transcatheter injection of the prepared emulsion into the lumen of the targeted vascular malformation. This technique and the specific nuances thereof—excluding the use of this novel embolotherapeutic agent—has previously been reported and expounded upon in previous publications of the current senior author (Fig 2, *C*)^8^^,^^9^ All procedures were performed under general anesthesia.

### Postoperative period

Postoperatively, all patients underwent a 24-hour observation; then followed as outpatients clinically at 2 weeks, 6 weeks, 6 months, and 12 months and evaluated for improvement of symptoms, reduction in lesion size, coagulopathic monitoring (in case of VMs), and development of side effects and complications. We also performed radiological follow-up with MRI and doppler ultrasound to assess changes in lesion volume and blood flow, respectively, starting at the 6-week clinical follow-up. Patients who required postoperative anticoagulation per our protocol (Fig 1) received it within 2 weeks postoperatively or baseline ambulation, whichever occurred first. Of note, we do not perform compression of the lesion following treatment so as to minimize sclerosant escape and nontargeted embolization.

### Reinterventions

Reinterventions were limited to those in whom a staged, multisession approach to DSE of the same extensive lesion was planned or anticipated prior to initiation of interventional therapy. If deemed necessary, these were performed no more frequently than every 4 weeks.

### Data collection and statistics

Data was collected via retrospective manual chart review. We collected demographics, lesion characteristics, technical success, clinical success, procedure complications, side effects, and re-interventions. Data analysis was done using SPSS software. Standard deviation was used as the measure of variance for our data. VRE had full access to all the data in the study and takes full responsibility for its integrity and the integrity of data analysis.

## Results

A total of 25 patients with LFVMs underwent 33 DSE with Yale OCR7737 between 2020 and 2023 (Tables I and II). Among these 25 patients, 10 (40%) were male and 15 (60%) were female. Most were of white race and non-Hispanic ethnicity (Table I). The mean age was 28 years, with a range of 1 to 70 years (Table I). Regarding lesion characteristics, 17 (68%) had VMs, while 8 (32%) had LMs. Most cases were isolated and nonsyndromic, with only 3 (12%) patients presenting with KTS. No other LFVM-related syndromes were identified. The most common site for LFVM involvement was the head and neck (48%), followed by the limbs (40%) and trunk (12%). After the DSE procedure was completed, patients had an average of 3.2 follow-up encounters, with the number of encounters ranging from 1 to 17 (Table I).

**Table I:** Characteristics of patients with low-flow vascular malformations (*LFVMs*) taken to direct stick embolization (*DSE*) with Yale OCR7737 between 2020 and 2023

Characteristic	No. (%)	Missing	Minimum	Maximum	Mean
Sex	25	0			
Male	10 (40)				
Female	15 (60)				
Age, years	25	0	1	70	28.4
Race	25	0			
White	13 (52)				
Black/African American	4 (16)				
Asian	1 (4)				
Native American	0				
Pacific Islander	1 (4)				
Unknown/not listed	6 (24)				
Ethnicity	25	0			
Non-Hispanic/Latino	21 (84)				
Hispanic/Latino	4 (16)				
Syndrome	25	0			
Isolated (nonsyndromic)	22 (88)				
Klippel-Trenaunay	3 (12)				
Location of LFVM	25	0			
Head and neck	12 (48)				
Trunk	3 (12)				
Limbs	10 (40)				
Type of LFVM	25	0			
Venous malformation	17 (68)				
Lymphatic malformation	8 (32)				
Post-DSE LIC	25	0			
Yes	13 (52)				
No	12 (48)				
DSE procedures per patient	25	0	1	5	1.4
Follow-up encounters per patient	25	0	0	17	3.2

*LIC,* Localized intravascular coagulopathy.

**Fig 3:**
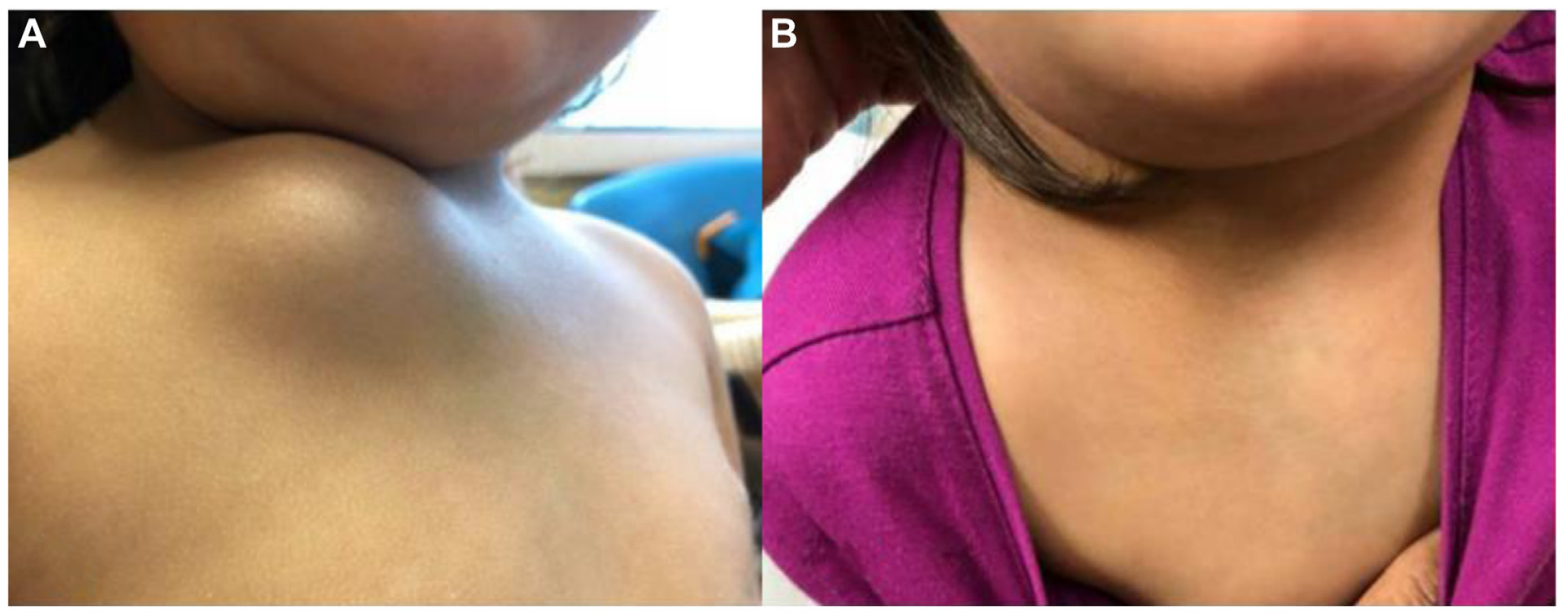
Decrease in lesion size in a patient after direct stick embolization (DSE) with Yale OCR-7737. A young patient with a mixed lymphatic malformation involving the anterior chest wall, inferior to the clavicle before **(A)** and after **(B)** DSE with Yale OCR7737.

**Fig 4:**
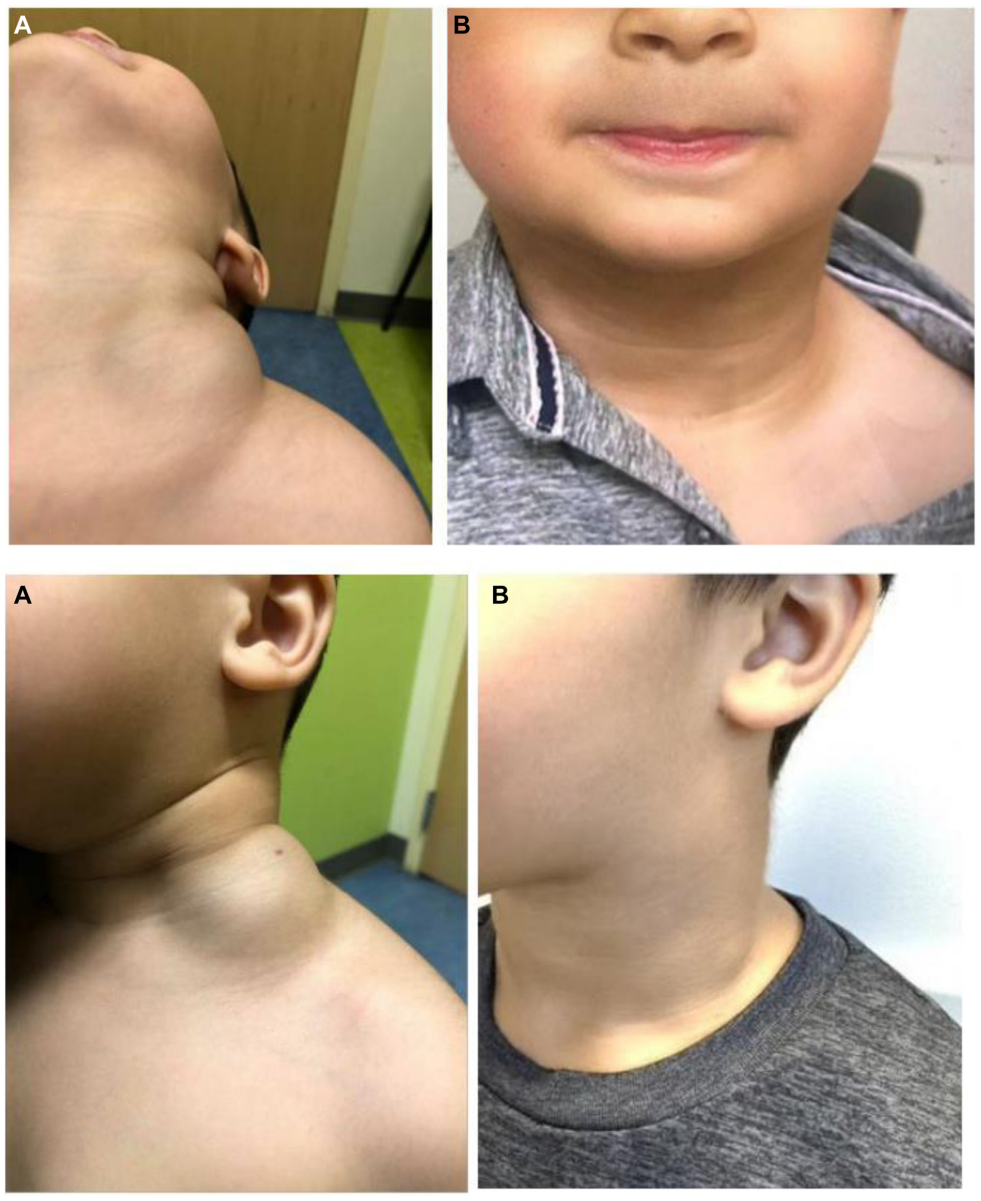
Change in lesion size in a patient after direct stick embolization (DSE) with Yale OCR-7737. A young patient with a multilobulated macrocystic lymphatic malformation involving the left lateral neck who underwent DSE with Yale OCR-7737. **(A)** Protuberant bulge associated with the lesion, worse with dependence and physical exertion before treatment. **(B)** Complete obliteration of the lesion with marked improvement in local disfigurement at the time of follow-up after direct stick embolization (DSE) with Yale OCR 7737. The lesion size is visibly reduced.

**Fig 5:**
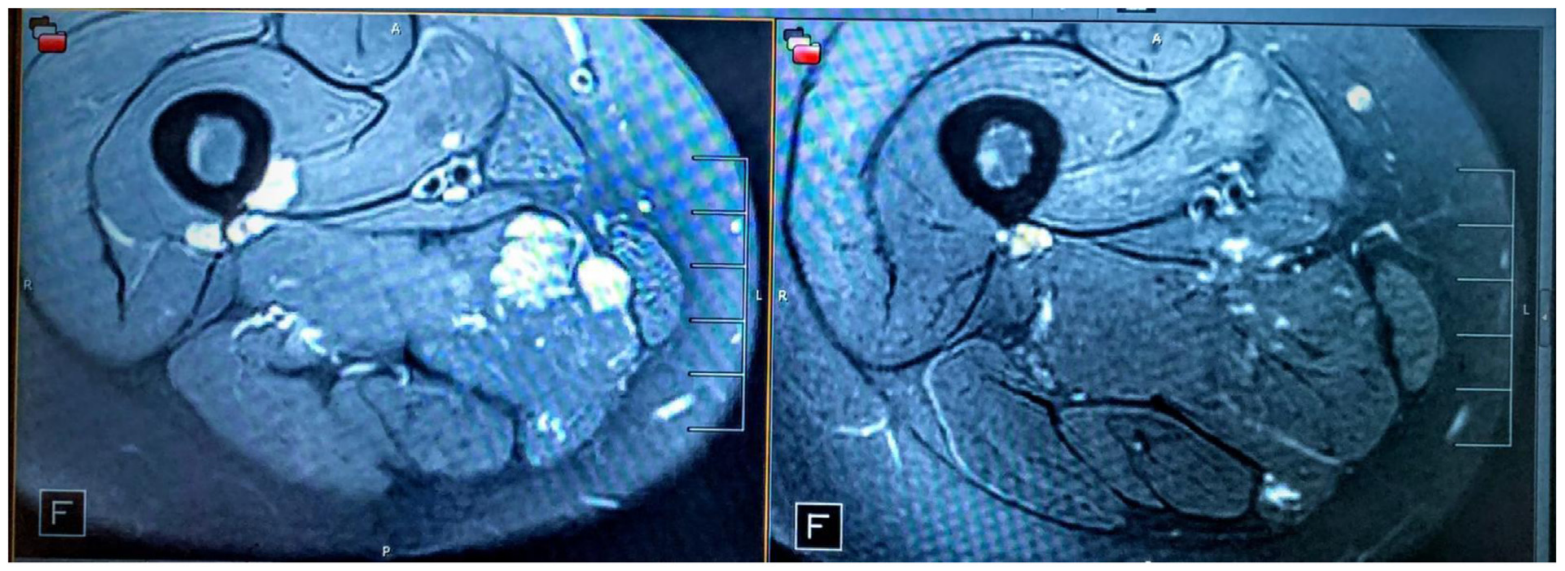
Change in lesion size seen on contrast-enhanced magnetic resonance imaging (MRI) after direct stick embolization (DSE) with Yale OCR-7737. Axial T2-weighted fat-suppressed MRI series performed 6 weeks after treatment of a right thigh intramuscular venous malformation with Yale-OCR7737 demonstrates markedly diminished lesion volume and enhancement.

**Fig 6:**
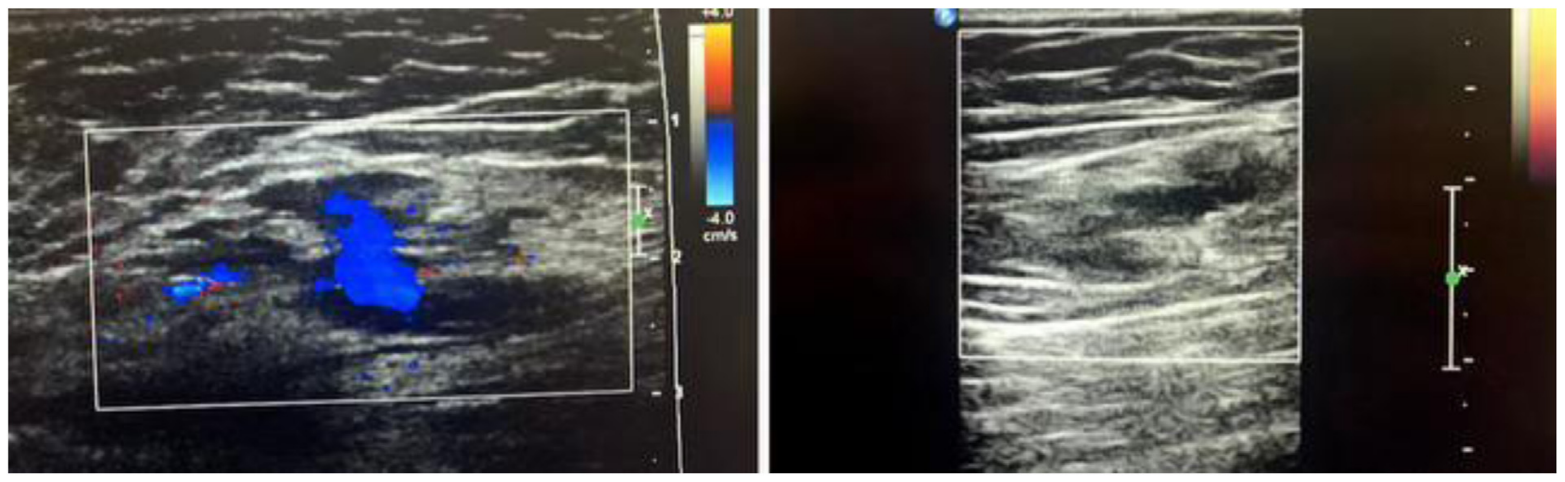
Change in intralesional blood flow seen on duplex ultrasound examination after direct stick embolization (DSE) with Yale OCR-7737. Duplex ultrasound examination of a lower extremity venous malformation before and after DSE with Yale-OCR7737. Color flow Doppler imaging performed 6 weeks after treatment demonstrates complete cessation of intralesional blood flow.

Technical success, defined as successful catheterization and intralesional delivery of the compound, was achieved in 100% of the cases (Table II). Clinical success, defined as improvement in signs and symptoms (requiring a two-clinician verification process, 1 surgical and 1 medical) with a radiologically documented reduction in flow and/or volume of the treated lesion, was also achieved in 100% of the cases (Table II, Fig 3, Fig 4, Fig 5, Fig 6).

**Table II:** Characteristics of direct stick embolization (DSE) procedures with Yale OCR7737 between 2020 and 2023

Characteristic	No. (%)	Missing
Aphthous ulcers^a^	33	0
Yes	7 (21.2)	
No	26 (78.8)	
Technical Success	33	0
Yes	33 (100)	
No	0	
Clinical success	30	3
Yes	30 (100)	
No	0	

a Patient developed aphthous ulcers after direct stick embolization with OCR 773.

No perioperative complications were reported. Side effects were limited to seven cases of transient, self-limited oral aphthous ulcers, accounting for 21.2% of all cases (Table II). Planned re-interventions were necessary in 8 of our 25 patients (32%). The mean number of DSE procedures per patient was 1.4, with a range of 1 to 5 DSE procedures per patient (Table I); a total of 17 patients (68%) underwent 1 DSE procedure, 7 patients (28%) underwent 2 DSE procedures, and 1 patient (4%) underwent 5 DSE procedures.

We assessed LIC in our patients, defining it as an elevated blood D-dimer with or without low fibrinogen. Preprocedural evaluation for LIC was conducted before 23 procedures, identifying LIC in 11 cases (47.8%) and ruling it out in 12 cases (52.2%) (Table III). Preprocedural LIC assessment was not done in 10 cases. Postoperative LIC assessment was conducted in all 33 cases, identifying 16 cases (48.5%) of LIC (Table III). Among these 16 cases of postoperative LIC, 9 (56.3%) had LIC before DSE, 4 (25%) did not have LIC before DSE, and 3 (18.7%) had no preprocedural LIC assessment.

**Table III:** LIC cases before and after direct stick embolization (*DSE*) procedures with Yale OCR7737 between 2020 and 2023

Characteristic	No. (%)	Missing
Preprocedural LIC	23	10
Yes	11 (47.8)	
No	12 (52.2)	
Postoperative LIC	33	0
Yes	16 (48.5)	
No	17 (51.5)	
LIC evolution in patients with preprocedural LIC	11	0
LIC improved after DSE	7 (63.6)	
LIC worsened after DSE	4 (36.4)	
LIC evolution in patients without preprocedural LIC	12	0
No LIC after DSE	8 (66.7)	
LIC after DSE	4 (33.3)	
Distribution of postoperative LIC cases	16	
Preprocedural LIC	9 (56.3)	
No preprocedural LIC	4 (25.0)	
No preprocedural LIC assessment	3 (18.7)	

*LIC,* Localized intravascular coagulopathy.

We also evaluated the evolution of LIC in these patients. LIC improvement was defined as a decrease in postoperative blood D-dimer compared with preprocedural levels, whereas LIC worsening was defined as an increase in postoperative blood D-dimer compared with preprocedural levels. Among the 11 cases with preprocedural LIC, LIC improved in 7 cases (63.6%) and worsened in 4 cases (36.4%) after DSE with Yale OCR-7737 (Table III). Among the 12 cases without preprocedural LIC, LIC did not develop after DSE in 8 cases (66.7%), whereas it did develop in 4 cases (33.3%).

## Discussion

We herein present the first-in-human, targeted, intralesional drug delivery via DSE with a sterile, intravenous-compatible mTOR inhibitor for treatment of LFVMs. Although still in its infancy, this limited experience is promising and has shown to be safe and effective. It warrants broader adoption, dedicated pharmacotherapeutic evaluation, and comparison with existing albeit off-label treatment options.

LFVMs are present at birth, do not regress spontaneously, and expand as the patient grows.^6^ Signs and symptoms can appear or worsen at any point in life; they are exacerbated by trauma, infection, or hormonal changes.^13^ The mean age of our patients was 28 years, with a wide range from 1 to 70 years, demonstrating the heterogeneity in the age of presentation of patients with LFVMs. This heterogeneity calls for treatment options to be evaluated across a wide range of age groups; patients of any age may require treatment for LFVMs. For this reason, we believe that the most effective and least toxic embolotherapeutic agent should be the agent of choice of these molecularly active lesions. Although the current study is far too small and the data presented therein merely retrospective, it nevertheless serves as a potential landmark study demonstrating the safety and efficacy of local intralesional mTOR inhibition as a standalone therapy for a variety of LFVMs.

Current DSE techniques using off-label embolotherapeutic compounds are rarely curative and carry a risk of serious adverse effects.^4^^,^^8^^,^^9^ Ethanol, which works via the destruction of endothelial cells, is by far the most effective sclerosant.^4^^,^^11^^,^^20^ Its efficacy, however, is counteracted by its potential for serious side effects, including local tissue toxicity, central cardiopulmonary toxicity, and death.^4^^,^^8^^,^^9^^,^^11^^,^^19^, ^20^, ^21^ Furthermore, none of the currently available embolotherapeutic compounds target the culprit mutated molecular pathway involved in the pathogenesis of LFVMs. In contrast, mTOR inhibitors directly target the pathogenesis of LFVMs. Initial animal models demonstrated rapamycin's effectiveness in delaying growth and decreasing lesion size of LFVMs.^13^, ^14^, ^15^, ^16^ Subsequent retrospective series and phase II trials in patients with LFVMs refractory to standard treatment further supported these observations, demonstrating the efficacy of oral sirolimus in improving pain, functionality, coagulopathy, lesion size, and quality of life in these patients.^12^^,^^17^^,^^18^ These studies also reported on the adverse effects of oral sirolimus, the most common being mucositis, bone marrow toxicity, hypercholesterolemia, and elevated liver enzymes.^12^, ^13^, ^14^, ^15^, ^16^, ^17^, ^18^ Preliminary results from the ongoing VASE trial, a phase 3 trial on the use of a 2-year regimen of oral sirolimus for the management of LFVMs, reported clinical improvement in 85% of patients, with recurrence of symptoms after therapy discontinuation in 54% of patients.^22^ These findings suggest that, although oral systemic mTOR inhibition is effective, it can lead to severe adverse effects and the recurrence of signs and symptoms upon discontinuation of therapy.

The proven efficacy of systemic mTOR inhibition as standalone therapy for LFVMs prompted us to seek a way of harnessing its benefits while minimizing its side effects. A new delivery route—local instead of systemic—seemed the best option to minimize systemic absorption and side effects, while allowing the drug to better work at the desired site. As such, our technique, which allows the direct intralesional delivery of an mTOR inhibitor, takes advantage of the proven clinical efficacy of oral systemic mTOR inhibition while avoiding the side effects and inconveniences associated with 2 times a day dosing, as well as the rebound exacerbation of lesions upon therapy cessation.

Current DSE procedures using off-label embolotherapeutic compounds have a clinical success rate of approximately 60% to 80%, with a risk profile that ranges from local ischemia to cardiovascular collapse and death.^6^^,^^8^^,^^11^^,^^20^^,^^23^, ^24^, ^25^, ^26^ This finding contrasts with our experience using Yale OCR-7737, which we found to be highly efficacious, with a technical and clinical success rate of 100%, and relatively safe, with a risk profile limited to transient aphthous ulcers. Although the most precise means of documenting radiographic improvement would have been through volumetric analysis of contrast-enhanced MRI studies before and after treatment, these secondary radiologic analyses and required personnel were not always readily available as there was no source of external funding for this particular series. This indeed is a limitation of the current study. However, the clinical success demonstrated by clinical images, crude MRI measurements, and color flow Doppler studies confirming diminished or absent intralesional flow, suggests our treatment was highly effective.

The average number of procedures per patient was low, with a mean of 1.4 procedures per patient and a range of one to five procedures per patient. Around 68% of patients did not require reinterventions, and among those who required reinterventions, seven of eight only required two reinterventions. Reinterventions were, in this experience, all planned based on operating surgeon preference and experience with more voluminous LFVMs, and with consideration given to minimizing potential hematologic derangements with aggressive single session embolization of larger lesions. The mean procedures per patient was lower than that reported for off-label embolotherapeutic compounds currently used for DSE, which is often determined to be approximately two to three interventions per patient.^23^, ^24^, ^25^ This finding suggests that, when compared with current DSE off-label embolotherapeutic compounds, Yale OCR-7737 may provide a higher rate of clinical improvement while requiring fewer interventions per patient. Although larger cohorts and longer follow-up periods are needed to substantiate these claims, it is reasonable to theorize that targeted drug delivery aimed at counteracting the culprit mutated pathway maybe a safer, more efficacious means of DSE as opposed to current techniques that use agents aimed at endothelial cell destruction and intraluminal inflammatory reactions. It is important to note that the treatment highlighted in this article, although indeed promising in many clinical regards, is far from curative as it stands currently and thus requires further development and study for maximal enhancement of clinical safety and benefit profile.

In our experience with Yale-OCR7737, side effects were limited to seven cases of transient aphthous ulcers that required no medical treatment, which account for 22.5% of all DSE procedures. This narrow side effect profile contrasts with the various side effects reported for the use of current DSE embolotherapeutic compounds and oral sirolimus. Current DSE embolotherapeutic agents have been associated with a vast array of side effects, which include skin ulceration, limb ischemia, and nerve injury, among others.^26^^,^^27^ Moreover, oral sirolimus is associated with multiple side effects, which include, but are not limited to, mucositis, asthenia, bone marrow toxicity, hypercholesterolemia, and elevation of liver enzymes.^14^, ^15^, ^16^^,^^22^ Our findings suggest that, although some systemic exposure is inevitable, foregoing repeated, twice daily dosing decreases dramatically the morbidity and toxicity of this therapy, without sacrificing clinical efficacy. This finding suggests that local targeting of the culprit mutated pathway sets of local endoluminal cascades that promote lesion regression and minimize lesion proliferation without the need for endothelial destruction or local inflammatory cascades, which has been the aim of current DSE methodology. Furthermore, should there be partial recurrence of the lesion during long-term follow-up, repeat DSE with OCR7737 can be performed in a manner that seems to be much less morbid and toxic than that which has been described with current techniques and is certainly more convenient and less toxic than twice daily dosing with frequent blood-draws and regular monitoring of blood levels.

Preprocedural assessment of D-dimer was conducted before 23 of the 33 DSE procedures. This evaluation identified LIC in 47.8% of these cases, a rate similar to that previously reported in other studies on LIC in VMs.^28^ In these cases, it is our practice to treat empirically with low-dose anticoagulant until 2 to 4 weeks after the procedure, and in select cases with severe coagulopathy and/or phlebolith formation, we consider long-term anticoagulation. Postoperative LIC assessment identified LIC in 48.5% of cases. The rates of LIC before and after DSE with Yale OCR-7737 were very similar. Further analysis showed that most patients without preprocedural LIC did not develop it after DSE with Yale OCR-7737, and most patients with preprocedural LIC saw an improvement in their D-dimer levels. Moreover, of the 16 cases of postoperative LIC, only 4 (25%) corresponded with patients who did not have LIC before DSE. All of this evidence suggests that DSE with Yale OCR-7737 does not lead to an increase in LIC cases. This finding is particularly interesting given that previous studies have shown that sclerotherapy with current embolotherapeutic agents, such as sodium tetradecyl sulfate, may lead to the development of LIC in patients with normal preprocedural D-dimer levels.^29^

The present study has various limitations, including its retrospective design, the lack of a control group to better assess efficacy and safety, the short period of follow-up for patients, and the lack of volumetric analysis of MRI imaging. Our experience with Yale OCR-7737, although promising, is limited and requires further analysis comparing its efficacy and safety to that of existing treatment options.

## Conclusions

To our knowledge, this study is the first in-human experience with direct intralesional delivery of an mTOR inhibitor for the treatment of LFVMs. This therapy provides the opportunity to directly target the mutated pathway responsible for the genesis of LFVMs in a localized fashion. Our experience with 33 DSE cases performed on 25 patients with LFVMs suggests that this therapy may be safe and highly effective for the management of these lesions. Our data also suggest that this therapy may not increase the risk of LIC in these patients, which has been associated with the use of embolotherapeutic compounds currently used for DSE. This new therapy seems to harness the benefits of mTOR inhibition while limiting its systemic toxicity, thus opening a new frontier in the interventional management of LFVMs.

## Declaration of generative AI and AI-assisted technologies in the writing process

During the preparation of this work, the authors did not use AI. The authors take full responsibility for the content of this publication.

## Author Contributions

Conception and design: VR, PP, NN

Analysis and interpretation: VR, NN

Data collection: VR, AL, SP, PP, NN

Writing the article: VR, NN

Critical revision of the article: VR, AL, SP, PP, NN

Final approval of the article: VR, AL, SP, PP, NN

Statistical analysis: VE

Obtained funding: Not applicable

Overall responsibility: NN

## Funding

None.

## Disclosures

N.N. is the co-founder and chief medical officer for Targeted Therapeutix, LLC.
